# In Search of the Origins of Consciousness: Simona Ginsburg and Eva Jablonka: *The Evolution of the Sensitive Soul: Learning and the Origins of Consciousness*. MIT Press, Cambridge, MA, 2019, 646pp, ISBN: 9780262039307

**DOI:** 10.1007/s10441-019-09363-x

**Published:** 2019-09-26

**Authors:** Jonathan Birch

**Affiliations:** Department of Philosophy, Logic and Scientific Method, London School of Economics and Political Science, London, UK

## Abstract

The Evolution of the Sensitive Soul is a landmark attempt to make progress on the problem of animal consciousness. Ginsburg and Jablonka propose a general cognitive marker of the presence of consciousness: Unlimited Associative Learning. They use this marker to defend a generous view about the distribution of consciousness in the natural world, on which a capacity for conscious experience is common to all vertebrates, many arthropods and some cephalopod molluscs. They use this inferred distribution to defend a view about the evolution of consciousness, on which it has evolved at least three times, first evolved at around the time of the Cambrian explosion (just over 500 million years ago), and was in fact the driving force behind that explosion. In this essay review, I reflect critically on the book’s central idea: the proposal that Unlimited Associative Learning provides a general marker of consciousness.

If I throw a ball across a field, there are facts about the physical processes going on as the ball flies through the air, but there is nothing it feels like from the ball’s point of view. The ball feels no joy or pain; it doesn’t experience the rush of the air or the colour of the sky. Now imagine a dog chasing the ball. There will again be facts about the physical processes going on as it sprints across the grass. But this time, there will also be *something it feels like from the dog’s point of view*. To be conscious in the most basic sense, or sentient, is to be like the dog rather than the ball. It is to be the kind of thing that has experiences, be they experiences of colour, shape, odour, pleasure, pain, joy or frustration. Somewhere in nature there is a line between the entities that have no experiences of any kind, and those entities that do have some experiences of some kind. Finding that line, and understanding how it was crossed, is a challenge for evolutionary biology.

It’s tempting to ask: how can we possibly understand the evolution of consciousness if we don’t understand the nature of consciousness? And isn’t understanding the nature of consciousness a notoriously hard problem? So hard it has come to be known as “the hard problem”? This didn’t deter many of the luminaries of nineteenth century evolutionary thinking, including Jean-Baptiste Lamarck, Herbert Spencer and George Romanes. But, with a few exceptions, it did deter most of the luminaries of twentieth century evolutionary thinking. For a long time, the received wisdom has been that evolutionary biology should remain silent on these matters, leaving them in the capable hands of philosophers and other full-time speculators.

Could it be that the time for silence has now passed? The last 30 years have seen the rise of a science of consciousness, aimed at uncovering the neural mechanisms involved in conscious experience, drawing on the technological advances of modern neuroscience. Opinions differ on how much progress the neuroscience of consciousness has made. The science is still often described as being in an immature or “maturing” phase (Wiese 2018; Michel et al. 2018). We don’t have a universally agreed set of signatures of consciousness or a standardised method for detecting them. Debate still rages as to how to look for consciousness in subjects who cannot verbally report their experiences, such as non-human animals and patients in a minimally conscious state. Yet it is widely agreed that the field has made at least some progress.

What sort of progress? Various potential neural signatures (or neural correlates) of consciousness have been identified (reviewed in Dehaene 2014, Chapter 4). A specific type of neural oscillation (the gamma wave) has long been thought to be important, although not everyone agrees about this. A specific type of event-related potential (the P3 wave) also seems to matter. Conscious experience seems to result not from localised brain activity in a specific region, but rather from brain activity that implicates many different regions of the cortex, as well as the thalamus. Some take the prefrontal cortex at the front of the brain to play a special role (Dehaene 2014), whereas others emphasize a “posterior hot zone” at the back of the brain (Boly et al. 2017).

The upshot is increasing optimism about the idea that science is ready to confront the puzzles of animal consciousness in a serious way, and to start making evidence-based assessments about which non-human animals are conscious and which are not (Boly et al. 2013; Le Neindre et al. 2017). This seems clearly correct in relation to non-human mammals. Our fellow mammals have a cortex like ours (albeit smaller), and the (thalamo)cortical mechanisms supporting consciousness in their brains are likely to be close homologues of those supporting consciousness in ours. It is a straightforward question whether those mechanisms are there or not, and the evidence so far strongly suggests that they are (Boly et al. 2013; Le Neindre et al. 2017).

Yet this strategy runs into a methodological problem as soon as we look beyond mammals, because non-mammals have no cortex. There are potentially homologous structures, such as the dorsal pallium in birds, but even in birds the differences are substantial (Güntürkün and Bugnyar 2016). The differences are greater still in reptiles, fish, and invertebrates. Does this mean non-mammals cannot be conscious? Of course not. It just means that, if they are conscious, the neural mechanisms supporting consciousness are likely to be quite different from those which support consciousness in human brains. But if non-mammals can’t report their experiences, and we can’t look for characteristic patterns of cortical activity, what exactly are we supposed to look for? What are the distinctive markers of consciousness in non-mammals? How can a science of non-mammalian consciousness get off the ground?

*The Evolution of the Sensitive Soul* is a landmark attempt to make progress on this problem. Ginsburg and Jablonka propose a general cognitive marker of the presence of consciousness: Unlimited Associative Learning. They use this marker to defend a generous view about the distribution of consciousness in the natural world, on which a capacity for conscious experience is common to “all vertebrates, many arthropods and some cephalopod molluscs” (p. 392). And they use this inferred distribution to defend a view about the evolution of consciousness, on which it has evolved at least three times in these three lineages, first evolved at around the time of the Cambrian explosion (just over 500 million years ago), and was in fact the driving force behind that explosion.

The curious title is a reference to Aristotle’s distinction between nutritive, sensitive (i.e. sentient) and rational souls, and this gives an initial flavour of the book as whole. Although packed with scientific detail, it also abounds with historical notes, fine quotations and illuminating digressions. The authors have a keen historical sensibility and a vast range of influences. Lamarck, Spencer, Romanes and William James feature particularly heavily. The result is a book that makes a hefty contribution to the literature, literally as well as metaphorically. The main text is 482 pages long, with 62 pages of notes and a 72-page bibliography with over 900 entries. The word “ambitious” does not do it justice. It would be a foolish enterprise for me to attempt to summarise it. What I will do instead is reflect critically on the book’s most important idea: the proposal that Unlimited Associative Learning provides a general marker of consciousness.

## Unlimited Associative Learning (UAL) as a Marker of Consciousness

At the heart of Ginsburg and Jablonka’s approach is the idea that, to study consciousness scientifically in non-mammals, we need cognitive markers of consciousness. If we look at behaviour alone, assuming that behaviour resembling human behaviour will have similar causes, we will be led down the path of unrigorous anthropomorphism. If we look at neuroanatomy alone, giving undue weight to the presence of a cortex, we will be led to an unjustified scepticism about consciousness in non-mammals. We need a middle path between credulity and undue scepticism, and the middle path is to look for cognitive markers.

Ginsburg and Jablonka argue, very plausibly, that we should look in particular at *learning*. When we ask what sorts of cognitive processing might be facilitated or enabled by conscious experience, learning is an obvious candidate, and the idea of a link between learning and consciousness has a long history (the book includes a quotation from Romanes positing such a link in 1883). Ginsburg and Jablonka are well aware, however, that not any kind of learning will do as a reliable marker of consciousness. There is evidence that a surprising amount of learning can occur even when the stimuli are not consciously perceived.

How sophisticated can unconscious learning be? One line of evidence concerns subliminal fear-conditioning in humans (Raio et al. 2012; Lipp et al. 2014). For example, Lipp et al. (2014) found that subjects can learn an association between a subliminally presented image (e.g. a picture of a wallaby) and an electric shock that occurs on stimulus offset, eventually showing conditioned electrodermal responses indicative of fear as soon as the image appears.^1^ Two more recent studies have found evidence of operant conditioning on subliminal stimuli (Pessiglione et al. 2008; Atas et al. 2014). Severely invasive experiments on rats by Grau et al. (reviewed in Allen et al. 2013) showed that spinally transected rats can do forms of conditioning and avoidance learning on electric shocks to the legs, below the point of transection. Information about these stimuli could not possibly reach the rat’s brain.

Reading all this, one starts to wonder: what’s left for consciousness to do? What more sophisticated forms of learning are there that might still be linked to consciousness? Here Ginsburg and Jablonka enter the fray with an interesting proposal. Their claim is that conscious experience is required for Unlimited Associative Learning (UAL), a form of associative learning with three distinctive features (pp. 230–232):

1. Compound stimuli: the conditioned stimulus can be a *compound* of discriminable perceptual features (e.g. a black-and-yellow buzzing object with a particular odour). These features may be in different sense modalities or in a single sense modality.
2. Novel stimuli: the conditioned stimulus can be *novel* to the animal, in the sense that it is “neither reflex eliciting nor preassociated” with an unconditioned stimulus or with past reinforcement (p. 232).
3. Second-order conditioning: there is *second*-*order* as well as first-order conditioning. A conditioned stimulus can be associated with some other conditioned stimulus or action, and so on, building up long chains of associative links between stimuli and actions.

Ginsburg and Jablonka’s thesis, in short, is that second-order conditioning involving novel, compound stimuli is a signature of consciousness. This kind of learning cannot happen, they claim, if the stimuli are not consciously experienced.

## The Inconclusive State of the Evidence

How good is the evidence for a link between consciousness and UAL? It’s fair to say that, to date, no experiment has convincingly shown the possibility of UAL on subliminal stimuli. The findings noted above all fall short of showing UAL without consciousness. For example, in the experiments on spinally transected rats, the conditioned stimulus (a small electric shock) was simple rather than compound, the stimulus was reflex eliciting rather than novel, and the conditioning was first-order. The Lipp et al. (2014) study described above comes close, because at least some of the images (e.g. the pictures of wallabies) were novel, compound visual stimuli. A recent study by Scott et al. (2018) also comes close: it purports to show unconscious learning of a cross-modal association between novel stimuli. The stimuli were a spoken name and a visual depiction of a profession. These are compound stimuli, because they consist of arrangements of perceptually discriminable elements (a name is a compound of phonemes; a picture is a compound of shapes and colours). At most, however, these two studies show that the first two components of UAL can be present without conscious awareness. They do not show any *second*-*order* conditioning of subliminal, novel, compound stimuli.

The hypothesis that UAL requires conscious awareness clearly deserves careful experimental scrutiny. It’s too soon, at this stage, to guess which way the inquiry will go. One possibility is that, as soon as psychologists look seriously for unconscious UAL, they will find it. This would not be the end of the story: it would refute the claim that conscious experience of stimuli is *necessary* for UAL, while still leaving open the possibility that conscious experience *facilitates* UAL, in the sense of boosting its reliability and/or speed or transforming its features in some other beneficial way. This would be the starting point for an investigation of how, if at all, conscious UAL differs from unconscious UAL.

Another possibility is that a learning ability even simpler than UAL will be shown to require consciousness. For example, I don’t know of any demonstration of the possibility of second-order conditioning *of any kind* on subliminal stimuli. This raises the intriguing possibility that second-order conditioning alone might already be a positive marker of consciousness, whether or not the stimuli are compound or novel. Second-order conditioning (but not on novel, compound stimuli) has been found in honey bees (Hussaini et al. 2007) and even snails (Papini 2010, p. 366). Yet I also don’t know of any experiments that have actively looked for second-order conditioning on subliminal stimuli in humans and failed to find it.

In short, the link between UAL and consciousness is promising yet largely uncharted territory. The hypothesis that UAL requires consciousness must be considered very tentative, since it has not been directly tested. At this point, a critic might remark: if we’re still awaiting strong evidence of a link between UAL and consciousness in humans, isn’t it premature to start using UAL as a positive marker of consciousness in non-human animals? And isn’t it even more premature to use the verdicts delivered by that marker as the basis for an account of the *evolution* of consciousness? If we do use it in this way, aren’t we just building conjectures on conjectures on conjectures?

I have some sympathy with this imagined critic, but I also have some sympathy with Ginsburg and Jablonka. They are trying to motivate a research programme, not to finish one. Their strategy is to make a bold but plausible conjecture about the type of learning that indicates consciousness, so as to get on with the task (in Part II of the book) of building a detailed account of the evolution of that type of learning in the context of the Cambrian explosion. This strikes me as a potentially fruitful approach, despite its inherently speculative character. The downside risk is not so bad: even if the link to consciousness does not survive scrutiny, they will still have produced a rich and interesting account of the evolution of associative learning.^2^

## The Taxonomic Distribution of UAL

The evidence regarding the distribution of UAL in the animal kingdom is also tantalizingly inconclusive. As noted above, Ginsburg and Jablonka say that the evidential picture we currently have justifies the attribution of UAL, and hence consciousness, to “all vertebrates, many arthropods and some cephalopod molluscs” (p. 392). I worry this exaggerates the strength of the current evidence, since direct experimental tests for the presence of UAL in non-human animals have not yet been done.

To get round this problem, Ginsburg and Jablonka count evidence of “proxies for UAL” (p. 383) as evidence of UAL, where the proxies include classical conditioning on compound stimuli, operant conditioning, and several other abilities the relation of which to UAL is unclear (numerical learning, navigation learning, and conceptual learning). Yet this only gives us evidence of *individual components* of the UAL package, not evidence of the whole package. The experiments described above give us reason to believe that, at least in humans, some individual components of the UAL package can occur without conscious awareness, including operant conditioning and classical conditioning on compound stimuli. Given this, it is crucial to establish the presence of the whole package.

Honey bees (*Apis* genus) provide an interesting case. They are among the most intensively studied of all invertebrates, and they are good learners. Honey bees can learn associations between novel, compound visual stimuli (Schubert et al. 2002) and, as mentioned above, they can also do second-order conditioning on olfactory stimuli (Hussaini et al. 2007). But can they do *second*-*order conditioning on novel, compound stimuli*? This is what UAL requires, but no one has ever looked for it. The evidence we do have regarding the individual components of UAL raises the probability that honey bees have the whole package, but it does not provide the kind of strong support that would be provided by a direct test for the presence of UAL. So, it seems premature to conclude that honey bees are probably capable of UAL, and the same could be said, I suspect, of any other invertebrate. Of course, this is compatible with thinking that UAL may ultimately be shown to be present in cephalopods and arthropods, if reliable, cheap, versatile tests for its presence can be developed. It just underlines the need for such tests.

## Conclusion

*The Evolution of the Sensitive Soul* is a book to capture the imagination of anyone with an interest in animal consciousness, whatever their disciplinary background. Neuroscientists, cognitive scientists, evolutionary biologists, comparative psychologists, and historians and philosophers of biology will learn a great deal from it. This is an exciting moment for animal consciousness research, with an interdisciplinary community of researchers starting to coalesce in a way reminiscent of the early days of the science of human consciousness. This emerging field needs foundational work: it needs people to put forward big ideas about the markers of consciousness and its distribution in the natural world. This book rises to the challenge, offering precisely the kind of big picture that the field needs. The idea at the heart of that picture—that Unlimited Associative Learning is a diagnostic marker of the presence of conscious experience, and one that is widespread in the animal kingdom—deserves further investigation. At present, the evidence is inconclusive.

## Footnotes

1 Earlier work (Soares and Öhman 1993; Esteves et al. 1994; Öhman and Soares 1998) had suggested these associations could only be formed when the image was “fear-relevant” (e.g. an image of a spider or a snake), but Lipp and colleagues’ results suggest the nature of the image does not matter.

2 The last chapter of the book presents an entertaining dialogue with an imagined critic, who makes a remark along these lines (p. 452).
